# Editorial

**Published:** 1861-05

**Authors:** 


### Editorial.

### CASE OF ALVEOLAR ABSCESS.

About the 15th of February last, Mr. B—— presented himsel for treatment of alveolar abscesses from the superior central incisors. His age was 26 years; he was of a cachectic constitution; had suffered for six years from malarious influences, thus the system became depraved; there was a pale, somewhat sallow complexion, indicating a want of red globules in the blood.

The gums, superior lip, and all that part of the face was very much swollen, and exceedingly sensitive; the soreness extended up along the nose on each side, though at first it was only on the left. The tumefaction indicated a large abscess about the root of the left central incisor; there was also an abscess of an older formation at the root of the right incisor; this however was not a point of tumefaction; the teeth were both sensitive to the touch, but the left far more so.

The anterior plate of the alveolar process was necrosed from the point of discharge through it down to its margin, and partially absorbed away; the gum over and above this was of a very dark purple color. The extraction of the teeth had been strongly urged, and the remark made that it was impossible to bring them to a healthy condition.

After a thorough examination of the case, proceeded to the treatment, the first step of which was to make a free incision through the gum, into the sack of the left incisor; when a free flow of fetid pus gushed out; washed out the sack first with warm water, then with a diluted tincture of arnica; and in a few hours after with the chlorid of soda. The frequent cleansing with such preparations was kept up for several days, till the swelling and soreness had much abated. A loosely rolled pledget of cotton was kept in the opening from the first; in order that the sides of the wound might not close together and prevent the escape of pus, this pledget extended to the bottom of the sack and protruded somewhat from the orifice, this was removed twice each day and the abscess thoroughly cleansed.

After the swelling had somewhat abated, creosote was added to the wash, the cotton was also moistened with this solution ; this was continued for several days ; then the pledgets moistened with pure creosote were introduced and changed every day. After the soreness had sufficiently abated, the pulp cavities were drilled into from the palatine surfaces of the teeth, and opened freely through the foramenæ at the points of the fangs, that the pus might escape, and that treatment might be employed through that channel also. These were cleansed out and the same applications made daily as by the opening in the gums. Creosote was pumped through the canals at each dressing, till it made its appearance through the opening in the gum ; then introduced into the canal entirely up to the foramenæ a loosely rolled pledget of cotton moistened with creosote, then placed a pledget, as already described, in the sack through the opening in the gum. Continued this treatment for about two weeks, occasionally alternating with tincture of iodine : at this time the fangs were filled with gold. Previous to the filling placed in the extreme part of the canal a small pledget of cotton, tightly rolled and moistened with creosote.

The treatment through the fistulous openings was continued for about two months. Creosote was used until the discharge of pus ceased ; after this iodine was used to promote granulation, which took place as rapidly as could have been anticipated with such a constitution. The opening on the left side has had no application for some time, (now the 25th of April,) and is almost entirely closed, and the gum of a perfectly healthy appearance about it ; the opening on the right side is still receiving treatment ; the gum about it appears perfectly healthy, granulations are closing the opening rapidly. The dead process was absorbed, and new process has been formed. The teeth are now quite firm in the sockets, as much so as their fellows. The teeth and gums, and the parts about them present as healthy appearance as though they had never been diseased. T.

### LARGE FILLINGS.

Making a very large filling, or building up the crown of a tooth, is almost always a protracted operation, and in some cases requires several hours of hard labor, more than can conveniently be endured by any operator ; it is also exceedingly irksome to the patient. This has been one great objection to this class of operations ; and again, it is in many cases impossible to prevent the encroachment of the saliva upon an operation of this kind long enough for its completion.

Many operators have the impression that if a case of this kind becomes moistened, or if the work is postponed for a time, that it can not be properly done thereafter. This is a false idea ; the work can be just as well performed, though it has been moistened, and remained in a half finished state for a day or a week, as though it had been kept perfectly dry, and the work not ceased from the beginning to the end.

We refer to this because we know that many operators, on this class of fillings, overwork themselves, and permit themselves to become exceedingly annoyed and fretted, when they are overtaken by the saliva. Every one knows that when he becomes wearied or fretted, and his patient fatigued or irritable, work should stop at once, and be postponed till the condition of both, operator and patient, is favorable, be that an hour or a week.

The objection to this course has been, that when a half made filling is subjected to the saliva, mucus and food, it becomes so vitiated that gold will not adhere to it.

If the gold already introduced has been properly adapted and consolidated, it will not absorb moisture, and all foreign substance upon its surface may be easily removed. Then, upon leaving a case of this kind in a half finished state, its surface should not be rubbed down or burnished, but remain as a serrated plugger leaves it; and when about to resume an operation, foreign substances may be removed from the gold, with a medium stiff tooth brush and warm water, or a little soap and water, and then clear water, or if there is much mucus, a little alcohol or chloroform may be used with the brush. Then dry with tissue paper or spunk ; then throw on a jet of warm air for a moment or two, and the gold will be found in just as good a condition for the reception of the subsequent portions, as though it had never been wet.

We oftentimes make two or three sittings in those large operations, and are confident that we make none the less perfect operations thereby, indeed far more perfect than could be made under excessive fatigue of both patient and operator. T.

### LANCING THE GUMS.

In the April number of the New York Dental Journal is an article on lancing the gums by Dr. Robertson, in which he takes to task Dr. J. D. White, the Dental Cosmos, and the Dental Register, and perhaps the rest of mankind. In copying an article from one journal to another, it is no evidence that the one who copies endorses every sentiment that may be contained in the selection. It is very frequently done because the article is good in the main, and inculcates some idea or principle that is important; and yet it may contain imperfections. We are sorry to see Dr. R. make such a quibble upon the "flap of gum."

We consider the separation of the gum from the neck of a tooth with the lancet before extraction, a matter of no small moment, Dr. R. and some others to the contrary notwithstanding. Dr. White stated some of the difficulties liable to attend the extraction of the teeth without first separating the gums. The objection which the opposers of this practice make, is, that it prolongs a painful operation. This in reference to a great many cases, is not true. It has been our lot to witness many of the operations of those who extract without lancing the gums, and often is the operation far more prolonged and more painful than it would have been had the proper separation been made.

For the most prompt and efficient performance of the operation, it is necessary that the instrument employed should make the most perfect embrace upon the tooth; now this adjustment can best be made when there is no impediment. The firm attachment of the gum to the neck of a tooth certainly constitutes an impediment. In adjusting a forceps upon a tooth, particularly a molar tooth, it is better to have the gum separated perfectly, so that the instrument may be passed without obstruction to its proper position, and slightly moved in various directions until the embrace is most perfect. It is frequently the case in those precipitate and forced adjustments, that the points or corners only of the forceps are in contact with the tooth, and by this means it is liable to be broken. It is impossible to attain that precise adjustment desirable when the attachment of the gum has to be torn up as the forceps is forced to its position. The teeth are far more liable to be broken, when the gum is not separated, the gum and soft parts are far more liable to be lacerated ; and when there is a hemorrhagic diathesis, bleeding is much more liable to occur, and more difficult to control when it does occur. The pain produced by separating the gum with a sharp lancet is very slight indeed, very much less than tearing it away with the blunt, rough point of a forceps. There are cases in which no cutting is necessary ; with these every one is familiar.

T.

### PROFESSIONAL JEALOUSY.

We observe a paper with the above heading in the April number of the New York Dental Journal, by Dr. John Allen. This paper is short, but strong and to the point ; indeed, he makes several points, yet we can hardly conceive that any dentist who is a gentleman, would be guilty of the practice to which he refers.

One, who we should find thus maligning those with whom he professes to be associated, we could hardly feel at liberty to receive as a professional brother.

The fact that one engages habitually in impugning the motives, or speaking ill to patients, of the operations of reputable dentists, is an evidence that he lacks the elements of true manhood, and of a gentleman, and those who are devoid of these qualities, we don't choose to recognize as professional brothers, however great their skill and knowledge in the profession may be ; still good men will occasionally be tempted to say injudicious things ; with them, however, it is not a practice.

Every dentist ought to know that, by speaking ill of his professional fellows, and especially of their operations, to patients, is the surest means of defeating his own object, unless he is with those devoid of common sense. It may be necessary sometimes to speak the truth about an inferior operation, but very seldom to the patient, for they are least able to understand the difficulties that attend the proper performance of many operations. It is more admissible to speak of them to a brother dentist, and most admissible to speak of imperfections to him who performed the work.

In all such cases, quite as much is conveyed by the manner as by the language. One may by language speak well of a matter, and at the same time, convey the most contemptible impression, by manner.

